# Packing Rearrangements in 4-Hydroxycyanobenzene Under Pressure

**DOI:** 10.3390/molecules24091759

**Published:** 2019-05-07

**Authors:** Ines E. Collings, Michael Hanfland

**Affiliations:** European Synchrotron Radiation Facility, 71 Avenue des Martyrs, 38000 Grenoble, France; hanfland@esrf.fr

**Keywords:** dipolar molecule, high-pressure single-crystal X-ray diffraction, phase transitions, intermolecular interactions, helium insertion, organic semiconductor

## Abstract

4-hydroxycyanobenzene (4HCB) is a dipolar molecule formed of an aromatic substituted benzene ring with the CN and OH functional groups at the 1 and 4 positions. In the crystalline state, it forms spiral chains via hydrogen bonding, which pack together through π−π interactions. The direct stacking of benzene rings down the *a*- and *b*-axes and its π−π interactions throughout the structure gives rise to its semiconductor properties. Here, high-pressure studies are conducted on 4HCB in order to investigate how the packing and intermolecular interactions, related to its semiconductor properties, are affected. High-pressure single-crystal X-ray diffraction was performed with helium and neon as the pressure-transmitting mediums up to 26 and 15 GPa, respectively. The pressure-dependent behaviour of 4HCB in He was dominated by the insertion of He into the structure after 2.4 GPa, giving rise to two phase transitions, and alterations in the π−π interactions above 4 GPa. 4HCB compressed in Ne displayed two phase transitions associated with changes in the orientation of the 4HCB molecules, giving rise to twice as many face-to-face packing of the benzene rings down the *b*-axis, which could allow for greater charge mobility. In the He loading, the hydrogen bonding interactions steadily decrease without any large deviations, while in the Ne loading, the change in 4HCB orientation causes an increase in the hydrogen bonding interaction distance. Our study highlights how the molecular packing and π−π interactions evolve with pressure as well as with He insertion.

## 1. Introduction

Organic semiconductors are promising materials for optoelectronic applications due to their tunable electronic properties, flexibility, and low cost [1,2,3]. Organic semiconductors usually contain π-conjugated motifs, allowing the formation of π-bonding orbitals with small energy gaps between the highest occupied and lowest unoccupied molecular orbitals (HOMO and LUMO, respectively) that facilitate charge transport [1]. For small molecule semiconductors, the charge mobility will depend on the packing of the π systems, determined through their intermolecular interactions. In this regard, structure–property relationships on single-crystal molecular semiconductors (avoiding grain boundary effects) is important for understanding the structural motifs that influence the charge mobility properties, allowing the design of improved semiconductors [1,2].

**Figure 1:**
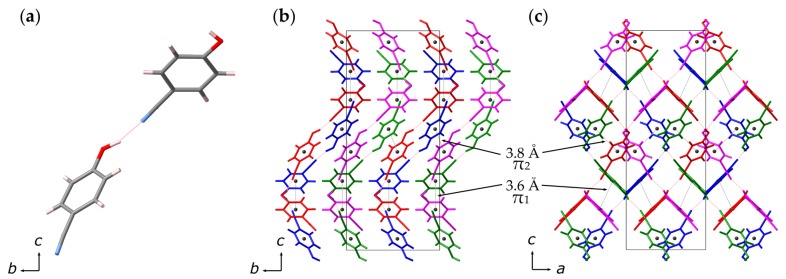
(**a**) Asymmetric unit of the two 4HCB molecules at ambient conditions. The colours represent the different atoms, where grey is C, red is O, blue is N, and pale pink is H. The hydrogen bonding is shown by the dotted red line. Extended structures of 4HCB with the hydrogen bonded connection of 4HCB molecules in one dimension is highlighted with different colours and viewed down the (**b**) *a*-axis and (**c**) *b*-axis. Benzene ring centroids are shown by the dark grey spheres, with the π−π interactions highlighted with dotted grey lines.

4-hydroxycyanobenzene (4HCB) is a dipolar molecule with both π−π and hydrogen-bonding interactions in the solid state and exhibits semiconductor properties (Figure 1) [4,5]. Interest in 4HCB stems from its three-dimensional charge transport properties and its easy growth as large single crystals [5,6,7,8]. The averaged charge mobilities along each crystallographic axis were measured as $\mu_{a}\approx 3\times 10^{-2}$, $\mu_{b}\approx 6\times 10^{-3}$, and $\mu_{c}\approx 4\times 10^{-6}$ cm${}^{2}$ V${}^{-1}$ s${}^{-1}$ and can be related to the crystal packing of 4HCB [6]. The lower charge carrier mobility along the *c*-axis can be explained from the lack of overlapping benzenic rings in this direction, while the *a*- and *b*-axes contain direct stacks of 4HCB molecules with distances of 9.2 Å and 10.7 Å separation [6]. π−π interactions are also found within the structure, although these are not aligned along a particular axis, but are also important in giving rise to the overall charge transport properties of 4HCB. The difference in charge mobility along the *a*- and *b*-axes can stem from (i) the longer distance between the benzene rings down the *b*-axis, (ii) only half of the benzene rings are oriented face-to-face along the *b*-axis, and (iii) the presence of the CN group in between the π-stacks down the *a*-axis. Due to these interesting properties, 4HCB has been suggested as a potential ionizing radiation detector [9,10].

4HCB crystallises in the Pbcn space group with the cell parameters *a* = 9.203(2) Å, *b* = 10.7370(10) Å, and *c* = 25.458(2) Å at ambient conditions [4,11]. The molecules form a spiral chain, governed by the hydrogen bonding interactions between the OH and CN groups found along the *c*-axis (Figure 1) [12]. The three shortest benzene ring centroid–centroid distances are observed at 3.64 Å (20${}^{\circ }$), 3.81 Å (21${}^{\circ }$), and 4.35 Å (19${}^{\circ }$ and 50${}^{\circ }$), with the values in the brackets indicating the angles between the ring normal and the vector of the ring centroids. The first two distances can indicate π−π interactions as they fall in the expected range of 3.3–3.8 Å and have a close face-to-face alignment [13]. The angle of ∼20${}^{\circ }$ between the π stacking indicates an offset which stabilises attractive π−σ interactions, increasing with the greater offset [13]. The 3.6 Å interaction (termed $\pi_{1}$) is between 4HCB molecules which are rotated by 90${}^{\circ }$ with respect to the other, while for the 3.8 Å interaction (termed $\pi_{2}$), the molecules are rotated by 180${}^{\circ }$.

High-pressure studies on molecular materials can give insight into the important intermolecular interactions, phase stability, and compressibility of the material [14,15,16,17,18,19]. Loss of aromaticity can also occur on compression by either polymerization reactions [20], or through the stabilization of one particular resonant state [21]. In this high-pressure study we aim to investigate whether better benzene packing can be achieved on compression, thus increasing the charge mobility capabilities of 4HCB. We also focus on how the hydrogen-bonding and π−π interactions are affected, which determine the overall packing of the 4HCB molecules.

We conducted two high-pressure single-crystal diffraction experiments on 4HCB with helium and neon as the pressure-transmitting medium (PTM), due to the unexpected entry of He into the structure of 4HCB. We report the anisotropic compressibility and phase transition behaviour of 4HCB. We find that the π−π interactions are much more affected by pressure than the hydrogen bonding interactions, and as 4HCB molecular orientations change on the phase transitions, new π−π interactions form. The hydrogen bonding interactions show a gradual decrease upon compression, with only a discontinuous increase on the phase transition of 4HCB in Ne. Finally, we suggest that the charge carrier mobility could be increased in the high-pressure phases due to the greater number of face-to-face packing of the benzene rings down the *b*-axis, as well as their closer distance.

## 2. Results

### 2.1. Compression in Helium

**Figure 2:**
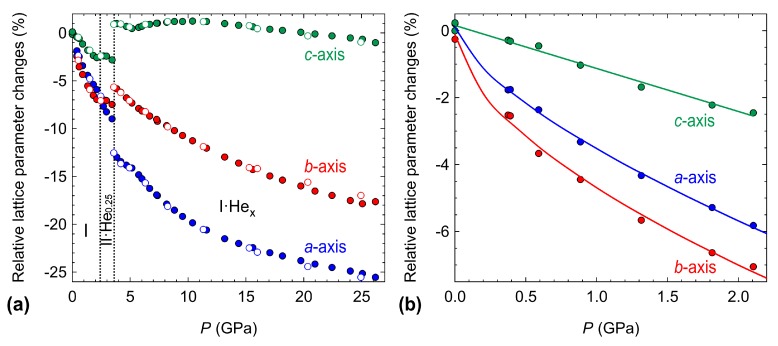
(**a**) Pressure dependence of the relative changes in lattice parameters of 4HCB with helium as the PTM. A zoom of the pressure dependence of the ambient phase up to 2.1 GPa is given in (**b**), with the solid lines representing the empirical fits for the *a*- and *b*-axes, and a linear fit for the *c*-axis. Decompression points are indicated by the open markers. Transition points are shown by the dotted black lines, and the different phases are indicated by I and II.

Compression of 4HCB was performed up to 26 GPa with He as the PTM. The lattice parameter data are shown in Figure 2, which highlights the stiffer *c*-axis compared to the *a*- and *b*-axes within the ambient phase. The compressibilities (*K*) along each axis are calculated as

(1) $$K_{l}=-\frac{1}{l}\frac{dl}{dp}$$

where l= lattice parameter and p= pressure. A linear fit to the lattice parameter pressure dependence up to 2.1 GPa gives $K_{a}=25.4\left(7\right)$ TPa${}^{-1}$, $K_{b}=28.4\left(15\right)$ TPa${}^{-1}$, and $K_{c}=12.8\left(12\right)$ TPa${}^{-1}$, as calculated in the webtool PASCal [22]. The anisotropy in the compressibilities are in agreement with the thermal expansion data [11], with the order of the softest to the stiffest axes as b>a>c. However, the pressure dependence of the *a*- and *b*-axes are not well represented with a linear fit, instead an empirical equation fits the curvature at low pressures much better, giving the compressibility range of $K_{a}$ = 34–19 TPa${}^{-1}$ and $K_{b}$ = 40–20 TPa${}^{-1}$ (Figure S1). Anisotropic changes in lattice parameters are very common in molecular materials due to the packing motifs and differing strengths of molecular interactions along different directions in the unit cell [14,23,24].

**Figure 3:**
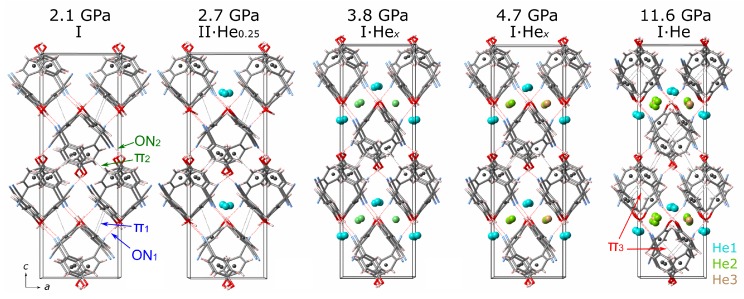
Structures of 4HCB compressed in He, with the coloured spheres representing the refined He positions on increase in pressure. The dotted grey lines indicate π−π interactions between the centroid positions of the benzene rings (dark grey spheres), and the dotted red lines show the O⋯N distances involved in hydrogen bonding interactions.

Two transitions were observed on compression of 4HCB with He as the PTM. The first at 2.4(3) GPa is associated with a change in symmetry from Pbcn to $Pbc2_{1}$, while the unit cell parameters remained quite similar. However, on refinement of the crystal structure, additional electron density was observed in between the molecular packing of 4HCB that could be refined as a He atom. The position of He atoms are found in between the columnar packing of the chains of 4HCB molecules (Figure 3). The symmetry lowering most likely occurs in order to keep the other symmetry-related site (in Pbcn symmetry) unoccupied with He. The position of the He atom does not correspond to the possible voids detected in Mercury at ambient conditions, which are in between the 3.6 Å π−π interactions down the *a*-axis (Figure S2). At 3.6(2) GPa, a transition from $Pbc2_{1}$ back to the original space group Pbcn is observed, which coincides with an increase in the He content by threefold. The position of He from the $Pbc2_{1}$ structure is retained (He1), and with the increase in symmetry, an extra symmetry-related site is populated. In addition to this, a second He site (He2) populates the positions in between the 3.6 Å π−π interactions, corresponding to the voids detected in Mercury at ambient conditions (Figure S2). At 4.7 GPa, a third He site is populated in between the He2 sites at a distance of 4 and 6 Å above and below, along the *b*-axis. From 4.7 GPa up to ∼10 GPa, the occupancy of the He3 site increases from 0.2 to 1. At 10 GPa, the loaded DAC was left at this pressure for a month, and could explain the extra disorder observed in the He2 site (split into two positions of half occupancy each) on re-measurement. This phase, 4HCB·He${}_{x}$, is stable up to the highest pressure measured of 26 GPa. Upon decompression, the helium content reduces and returns back to zero below 2.5 GPa, within the ambient phase regime. Phase II is not observed on decompression, which could be due to the very narrow pressure range that it is stable in.

**Figure 4:**
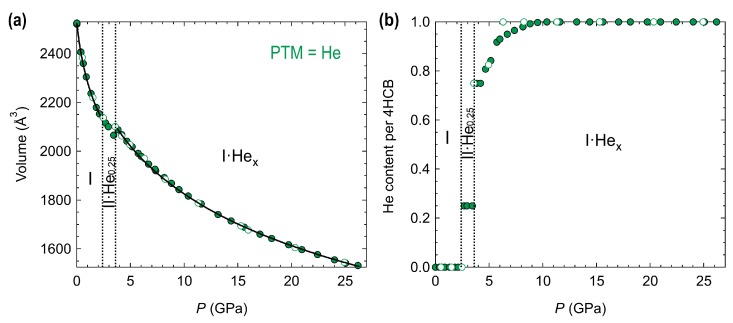
Pressure dependence of (**a**) the unit cell volume, with the solid black lines indicating third-order Vinet equation of state fits, and (**b**) the helium content in the unit cell per 4HCB molecule (we note that there are 16 4HCB molecules in the unit cell). Decompression points are indicated by the open markers and the transition points are indicated by dotted lines.

Third-order Vinet equations of state (EOS) were used to fit the volume pressure dependence for the 4HCB compound compressed in He using EoSFit [25] (Figure 4). The bulk modulus ($B_{0}$), its derivative ($B^{\prime }$), and the zero-pressure volume ($V_{0}$) are given in Table 1. For the high-pressure phase with He (I·He${}_{x}$), only the range from 10.4–26 GPa was used for fitting as it had a stable He content (Figure 4b). For the fitting procedure in phase I·He${}_{x}$, the parameters $V_{0}$, $B_{0}$, and $B^{\prime }$ could not be refined at the same time, otherwise the values would diverge. So the $B^{\prime }$ was first refined after keeping $V_{0}$ and $B_{0}$ fixed at the estimated value, and once refined, the $V_{0}$ and $B_{0}$ parameters were refined while keeping $B^{\prime }$ fixed. This procedure was repeated several times, where two parameters could be refined at the same time until convergence. The volume changes on the incorporation of He into 4HCB at 2.7 GPa (phase I to II·He${}_{0.25}$) and 3.4 GPa (phase II·He${}_{0.25}$ to I·He${}_{x}$) is estimated to be +0.7% and +2.4%, respectively, based on the extrapolation of the EOS fits (Figure S5).

The bulk modulus of the ambient phase, $B_{0}$ = 6.0(6) GPa, is similar to the ones found for other six-membered ring molecules such as benzene (5.5 GPa) [26], aniline (5.4 GPa) [27], and triazine (6.3 GPa) [28]. 4HCB·He${}_{x}$ has a bulk modulus that is nearly twice the value of the ambient phase ($B_{0}$ = 11.1(3) GPa), which can be explained by the denser packing of the 4HCB molecules and by the presence of He atoms.

**Figure 5:**
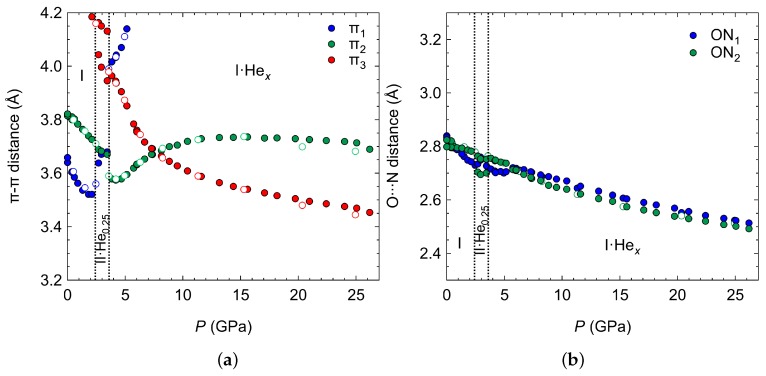
(**a**) The π−π distances on compression between the two initially parallel 4HCB molecules (which are symmetry related in the Pbcn phase), where the blue and green markers indicate the 3.6 and 3.8 Å π−π interaction, respectively at ambient and the red markers are for the new interactions that form within the square-shaped columns as indicated in Figure 3. (**b**) O⋯N hydrogen bonding distances on compression, where the colour of the markers indicate the same direction of H-bonding as the π−π interactions.

Within the ambient phase, the π−π distances decrease rapidly (Figure 5a), although the angle between the normal of the benzene ring and the centroid–centroid vector increases also, suggesting that the molecules evolve towards a more staggered arrangement with increasing pressure (Figure S3). Upon the He insertion, the $\pi_{1}$ interaction is perturbed the most, and increases beyond 4 Å, which is due to the He residing in the space between the molecular packing that is connected via this π−π separation (Figure 3). Instead, a new π−π interaction ($\pi_{3}$) begins to be stabilised above 5 GPa within the columnar packings as viewed down the *b*-axis. Even though there are no He atoms residing in between the molecular clusters connected via the $\pi_{2}$ interaction, it is also perturbed by the insertion of He, but its distance on compression still remains lower than its initial starting value (Figure 5a). The hydrogen bonding interactions steadily decrease on compression, even those that are located along the molecular clusters with He in between. This highlights the much stronger hydrogen bonding interactions compared to the π−π.

### 2.2. Compression in Neon

**Figure 6:**
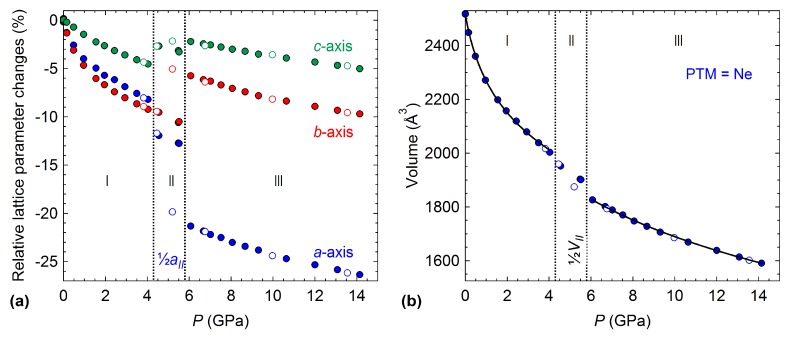
Pressure dependence of (**a**) the relative changes in lattice parameters of 4HCB with neon as the PTM, and (**b**) the unit cell volume, with the solid black lines indicating third-order Vinet equation of state fits. Transition points are shown by the dotted black lines, and the different phases are indicated by I, II, and III. The *a*-axis and the volume have been halved in phase II to be comparable to phases I and III. Decompression points are indicated by the open symbols.

**Figure 7:**
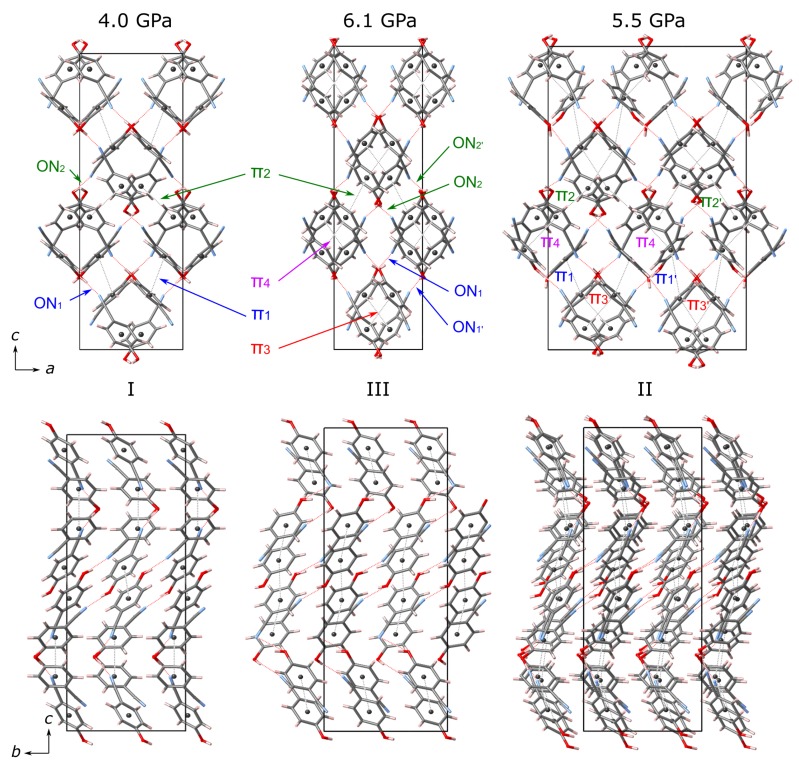
Structures of 4HCB compressed in neon for the ambient (**I**) and second high-pressure phases (**III**) with their intermolecular interactions indicated for possible π−π and hydrogen bonding (ON) interactions. The labels and colours of the indicated interactions relate to the same markers used in Figure 8. The first high-pressure phase (**II**) is also shown on the right. The dotted grey lines show possible π−π interactions between the centroid positions of the benzene rings (dark grey spheres), and the dotted red lines show the O⋯N distances involved in hydrogen bonding interactions.

Due to the insertion of helium into the 4HCB structure on compression, the experiment was repeated with neon as the PTM. The larger radius of neon should prevent its entry within the crystal structure on compression [29]. The changes in unit cell parameters and volume with compression are shown in Figure 6. The ambient phase of 4HCB is now stable up to 4 GPa (compared to 2.4 GPa when compressed in He). The same anisotropic compression of the lattice parameters is observed as when compressed in He, where the *c*-axis is the least compressible. Above 4 GPa, 4HCB transforms to a new phase (II) with a doubling of the *a*-axis and a reduction in symmetry from the ambient space group Pbcn to $Pbn2_{1}$ (this setting was chosen due to its similarity to the ambient phase). The main structural changes associated with this transition are the orientations of the 4HCB molecules along the π−π interactions (Figure 7). In particular, one of the symmetry-unique 4HCB molecules begins to change orientation, but not all at once, causing a doubling of the *a*-axis as half of the 4HCB orientations change. In this phase (II), the molecules viewed down the *a*-axis are now slightly staggered, instead of directly stacking as known in the ambient phase. The 4HCB-II phase is only stable up to 5.8(3) GPa, after which it transforms to a third phase (III) with the space group symmetry $Pbc2_{1}$. In 4HCB-III, the doubling of the *a*-axis disappears, returning to the original unit cell setting of the ambient phase, as well as a direct stacking of 4HCB molecules down the *a*-axis. A similar orientation of all 4HCB molecules is now adopted with a greater face-to-face packing down the *b*-axis. The 4HCB-II phase can be considered as an intermediate phase of the transformation of I to III, due to the intermediate orientations observed for the 4HCB molecules. Moreover, its formation may have been influenced by the solidification of neon at 4.8 GPa [30]. Upon decompression, all transitions were reversible, with some hysteresis observed for the phase III to II transition.

The volume dependence of the 4HCB-I and 4HCB-III phases were fitted with a third-order Vinet equation of state, giving bulk modulus values of $B_{0}^{I}=5.5\left(2\right)$ GPa and $B_{0}^{\mathrm{III}}=9.7\left(2\right)$ GPa, respectively. By extending the EoS fits into the pressure region of phase II, volume discontinuities on the different transitions could be estimated. The first transition from phase I to II is associated with a volume discontinuity of −1.7% (calculated at 4.6 GPa), while the phase II to III transition gives rise to a −2.7% volume change (calculated at 5.5 GPa), as shown in Figure S6. The stiffening of the high-pressure phase is also accompanied by a reduction in the derivative of the bulk modulus. The ambient $B_{0}$ values for both He and Ne loadings are the same within the error, while the high-pressure phases do show a slightly higher $B_{0}$ for 4HCB·He, presumably originating from the increased stiffness when He is inside the structure (Table 1). The stiffer HP phase compared to the ambient phase can be linked to a denser packing of the 4HCB molecules, as well as from the change in the π−π interactions.

Upon the transition to the 4HCB-III phase, we can clearly observe a discontinuous change in the orientations of the 4HCB molecules that are involved in the shortest $\pi_{1}$ interaction. This leads to an increase in the centroid–centroid distance of the benzene rings to 4.4 Å and instead new π−π interactions are stabilised on the transition ($\pi_{3}$ and $\pi_{4}$), within the columnar packings of the 4HCB chains, as viewed down the *b*-axis (Figure 7). The stacking angles between the 4HCB molecules, defined as the angle between the benzene ring normal and the centroid−centroid vector, discontinuously change on the transition from I to III and evolve towards similar values (∼30−37${}^{\circ }$) (Figure S4). The greatest reduction in stacking angle is observed from the originally large $\pi_{3}$ distance of 4.4 Å that changes to 3.6 and 3.5 Å in the HP phase, forming the new $\pi_{3}$ and $\pi_{4}$ interactions, respectively. The 4HCB-II phase contains a mixture of the π−π interactions from phases I and III, supporting its role as an intermediate phase.

**Figure 8:**
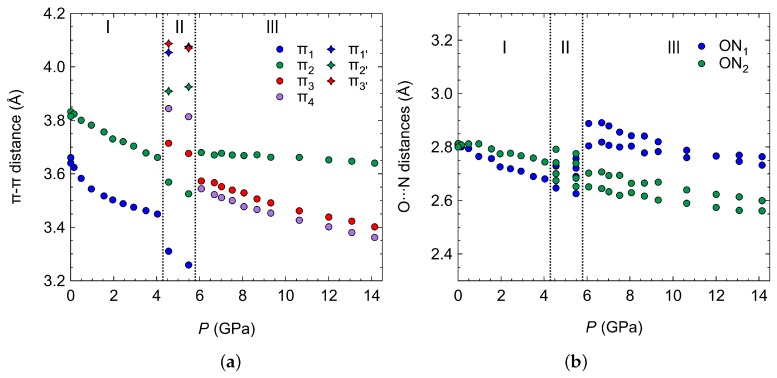
(**a**) The π−π distances on compression between the two initially parallel 4HCB molecules (which are symmetry related in the Pbcn phase), where the labels indicate the interactions depicted in Figure 7. (**b**) O⋯N hydrogen bonding distances on compression.

The O⋯N distances involved in hydrogen bonding decrease steadily on compression within the ambient phase, but show a discontinuous change on the I to III phase transition (Figure 8). This can be explained by the change in π−π interactions, where loss of the $\pi_{1}$ interaction leads to longer O⋯N distances in the same direction (ON${}_{1}$). This highlights that packing efficiency and formation of favourable π−π interactions are preferred over conserving the stronger hydrogen bonding interactions at a given distance after a critical pressure point. In the 4HCB-II phase, we observe a broader range of O⋯N distances due to the many symmetry-inequivalent 4HCB molecules present in the unit cell, and the intermediate nature of this phase.

## 3. Discussion

Within the ambient phase, the compression of 4HCB showed some degree of anisotropy in both PTMs used, as is known in its thermal expansion behaviour [11]. In particular, the *a*- and *b*-axes compressed similarly, while the *c*-axis was much stiffer. This can be rationalized by the hydrogen bonding interactions found along the *c*-axis, strengthening this axis. The compression behaviour of 4HCB in He and Ne was different beyond 2.4 GPa due to the entry of He into the 4HCB structure at this pressure. This had several consequences: (i) phase transitions at much lower pressure, (ii) disruption of the shortest $\pi_{1}$ interaction, and (iii) a pressure-dependent behaviour that was highly influenced by the content of He in the structure. Entry of He in non-porous structures on compression is a phenomenon that has been observed in Arsenolite [31,32,33], CaZrF${}_{6}$ perovskite [34], and dimethylammonium metal formates [35]. The compression of 4HCB with Ne as the PTM gives insight on how 4HCB behaves under pressure (instead of 4HCB·He${}_{x}$). A phase transition is initiated at 4.3 GPa by going through an intermediate phase with a doubled cell, until reaching the high-pressure phase III at 5.8 GPa. The transition involves significant changes in the orientation of the 4HCB molecules, causing a rearrangement of the π−π interactions. This rearrangement could allow for greater charge mobility along the *b*-axis as twice as many direct 4HCB stacks are formed. Upon increase in pressure, the stagger between 4HCB molecules increases, but converge to similar values in the 4HCB-III phase at a range of ∼30−37${}^{\circ }$, while the stagger between 4HCB stacking in 4HCB·He${}_{x}$ continuously increases in the range of ∼25−45${}^{\circ }$. This change in orientation does not significantly affect the hydrogen bonding distances in the case of 4HCB compressed in He, which continue to decrease linearly. In contrast, the 4HCB orientation change in 4HCB-III, which is much more dramatic, causes an increase in the H-bonding distances along the same direction.

## 4. Conclusions

In conclusion, we have investigated the molecular packing and intermolecular interactions of 4HCB at high pressure with two separate loadings in He and Ne. Both experiments showed the presence of two phase transitions, but the inclusion of He into 4HCB gave a different phase behaviour, which was mainly dominated by the increasing inclusion of He. The changes in intermolecular interactions was also slightly different in the two loadings, in particular the high-pressure phase of 4HCB in Ne (III) yielded three similar π−π interactions with staggered parallel packing, while in 4HCB·He${}_{x}$, a larger range of stagger between π stacking is observed. The high-pressure phases, in particular 4HCB-III, have a greater face-to-face packing down the *b*-axis, which could have implications for the charge mobility capabilities of 4HCB.

## 5. Materials and Methods

**Figure 9:**
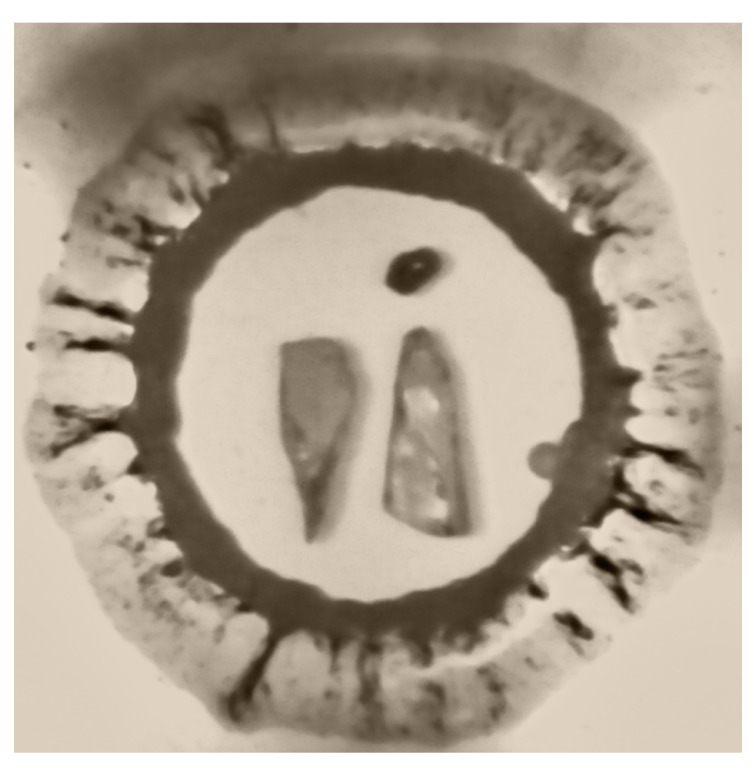
Loading of 4HCB in He at 6.7 GPa that show the two single crystals of 4HCB, the ruby, and the tungsten.

4-hydroxycyanobenzene (4HCB) was purchased from Sigma-Aldrich (purity 95%) and re-crystallised from a saturated diethyl ether solution by slow evaporation at room temperature [8]. Two separate loadings of 4HCB were performed using helium and neon as the pressure-transmitting medium. For both loadings, membrane driven LeToullec type diamond anvil cells, equipped with Boehler-Almax anvils were used and stainless steel was employed as the gasket material. For the first loading with helium as the pressure-transmitting medium (PTM), two single crystals of 4HCB were used, both with dimensions of 90 × 40 × 10 μm. The compression run up to 26 GPa and decompression down to 15.6 GPa used crystal 1, and subsequent decompression points used crystal 2 as crystal 1 had broken into many pieces (possibly from the diffusion of He). In the second loading with neon as the PTM, three single crystals of 4HCB were loaded with dimensions 90 × 50 × 17 μm (c3), 50 × 40 × 20 μm (c4), and 40 × 20 × 15 μm (c5). Crystal 3 was used for compression up to 15 GPa and decompression down to 4.8 GPa, after which the crystal broke in two, and subsequent decompression points used crystal 4. Together with the crystals, a ruby for pressure calibration and a small piece of tungsten for centering purposes were included in the sample chamber (Figure 9).

High-pressure single-crystal X-ray diffraction on 4HCB was measured at the ID15B beamline of the European Synchrotron Radiation Facility, Grenoble up to 26 GPa and 15 GPa for the loadings with helium and neon, respectively, using monochromatic X-ray radiation (λ = 0.4111 Å). Diffraction patterns were collected with a Mar555 flat panel detector using steps of 0.5${}^{\circ }$ oscillations over a ±38${}^{\circ }$ω scan range about the vertical axis. Pressures were determined using the ruby fluorescence method before and after each diffraction measurement. Lattice parameter determination and integration of the reflection intensities were performed using the CrysAlisPro software [36]. Refinement of the ambient structure was performed using SHELXL within the graphical user interface ShelXle [37,38]. SHELXT was used for structure solution of the new high-pressure phases [39]. The location of He atoms could be observed in the Fourier difference maps. H atoms of the benzene ring were added using geometric constraints. The H atoms of the hydroxyl group were located using the Fourier difference maps if applicable and distance restraints were applied to the O atom. Therefore, we note that the position of the H from the hydroxyl group is not well constrained and may not be very accurate for all pressure points. This is why all our hydrogen bonding interactions were interpreted via the O⋯N distances. In the 4HCB-II HP phase, and in two of the structure refinements of the III phase, distance restraints for H⋯N had to be added in order to obtain a reasonable orientation of the hydroxyl group. The thermal parameters of the H atoms were constrained as 1.2 times those of the atoms they were connected to.

## Figures and Tables

**Table 1:** The bulk modulus ($B_{0}$) and its derivative ($B^{\prime }$), and the zero volume pressure ($V_{0}$) parameters obtained from the third-order Vinet equation of state. Please note that the errors for the high-pressure ranges are only based on two parameters refining at one time, and the highest errors of the two combinations were selected.

PTM	*P* Range (GPa)	$B_{0}$	$V_{0}$	$B^{\prime }$
He	0–2.1	6.0(6)	2525.3(3)	10.0(14)
He	10.4–25.1	11.1(3)	2520(15)	6.65(10)
Ne	0–4.0	5.5(2)	2516.7(10)	11.0(4)
Ne	6-14	9.7(2)	2377(10)	6.78(10)

## Supplementary Materials

The following are available online, Figure S1: Calculated compressibilities, Figure S2: Voids calculated in Mercury. Figure S3: Angles between the benzenic ring normal and the centroid–centroid vector for 4HCB compressed in He, Figure S4: Angles between the benzenic ring normal and the centroid–centroid vector for 4HCB compressed in Ne, Figure S5: Volume discontinuities for the transitions in He, Figure S6: Volume discontinuities on the transitions in Ne, Table S1: Variable-pressure lattice parameter data for compression in He, Table S2: Variable-pressure lattice parameter data for compression in Ne. The pressure-dependent structures of 4HCB in both He and Ne loadings have been deposited to the CCDC with the numbers 1905777–1905833, and cif files are also provided as supplementary.
