# Supplementary material for: Packing Rearrangements in 4-Hydroxycyanobenzene Under Pressure
Source: Molecules. 2019 May 7;24(9):1759. doi: 10.3390/molecules24091759 (PMC6539840; doi:10.3390/molecules24091759)
Supplement: Supplementary file 1 [file molecules-24-01759-s001.pdf]

Supplementary Materials: Packing rearrangements in 4-hydroxycyanobenzene under pressure

Ines E. Collings [1,*] and Michael Hanfland [1]

[1] European Synchrotron Radiation Facility, 71 Avenue des Martyrs, 38000 Grenoble, France.
* Email: ines.collings@esrf.fr.

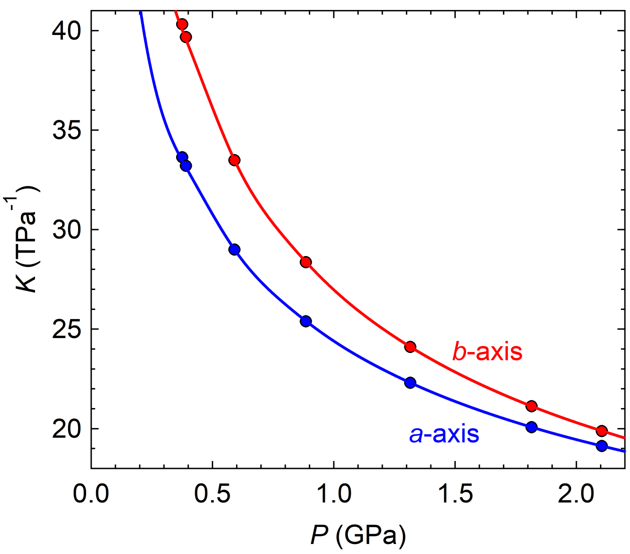

Figure S1. Calculated compressibilities obtained from the empirical fits of the *a*- and *b*-lattice parameter pressure dependence from PASCal [S1].

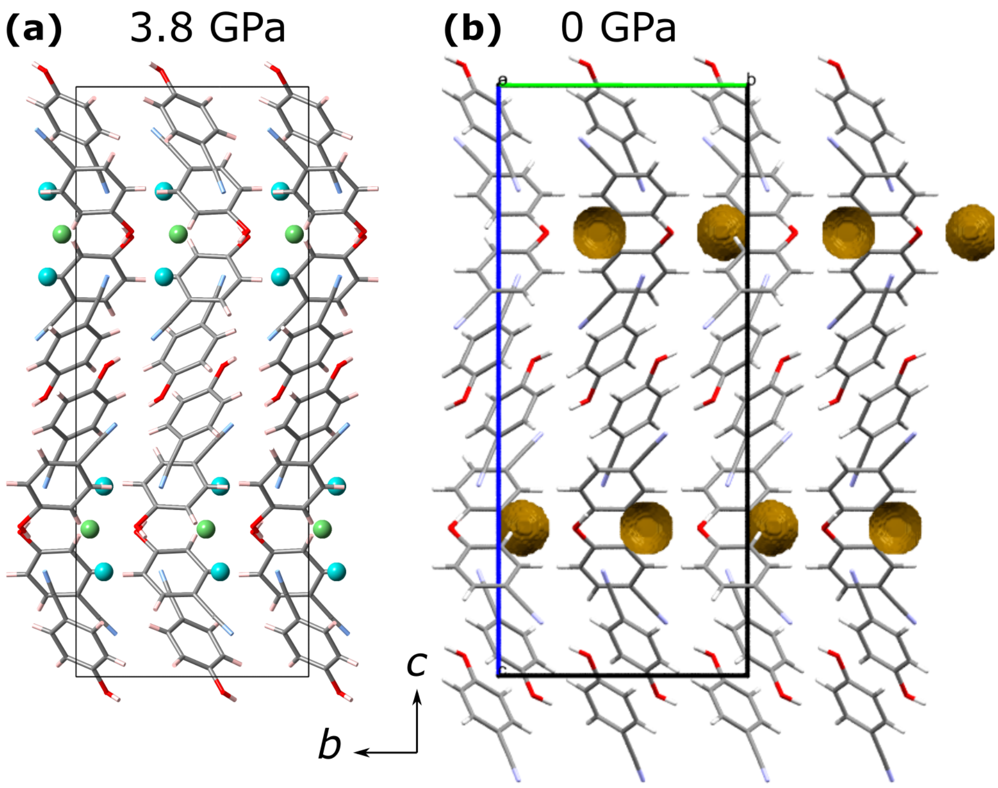

Figure S2. (a) Structure of 4HCB at 3.8 GPa with the He sites shown in blue (He1) and green (He2) atoms. (b) Voids calculated in Mercury for the ambient 4HCB structure using the radius of He as 1.07 Å,[S2] with a grid spacing of 0.1.

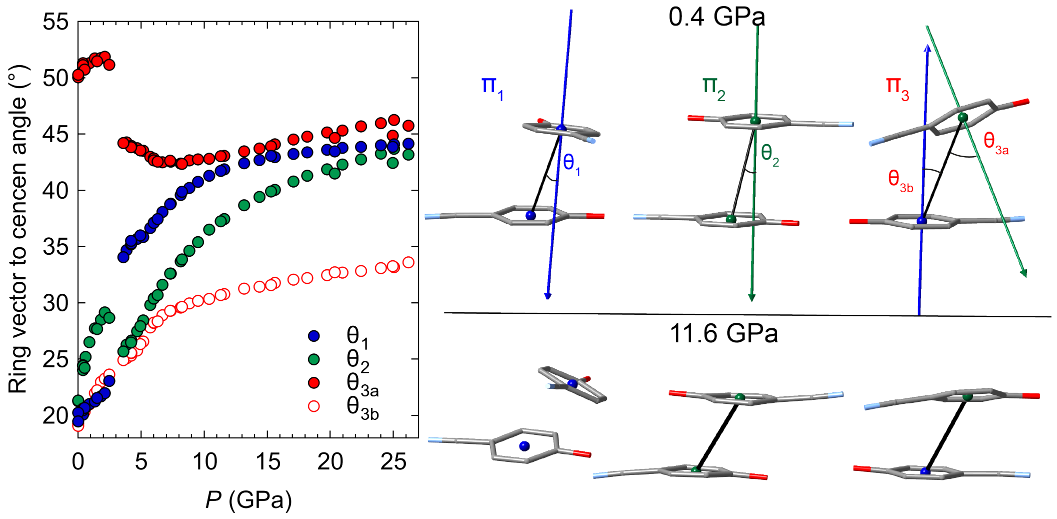

Figure S3. Angles between the benzenic ring normal and the centroid–centroid vector in 4HCB compressed in He as a function of pressure. The angles are illustrated for the three different combinations of interactions: centroid1–centroid1 (π_1), centroid2–centroid2 (π_2), and centroid2–centroid1 (π_3), shown at 0.4 GPa and at 11.6 GPa. The benzenic ring vectors are shown by the coloured arrows for the packings at 0.4 GPa. The abrupt changes in 4HCB orientations are observed upon He insertion.

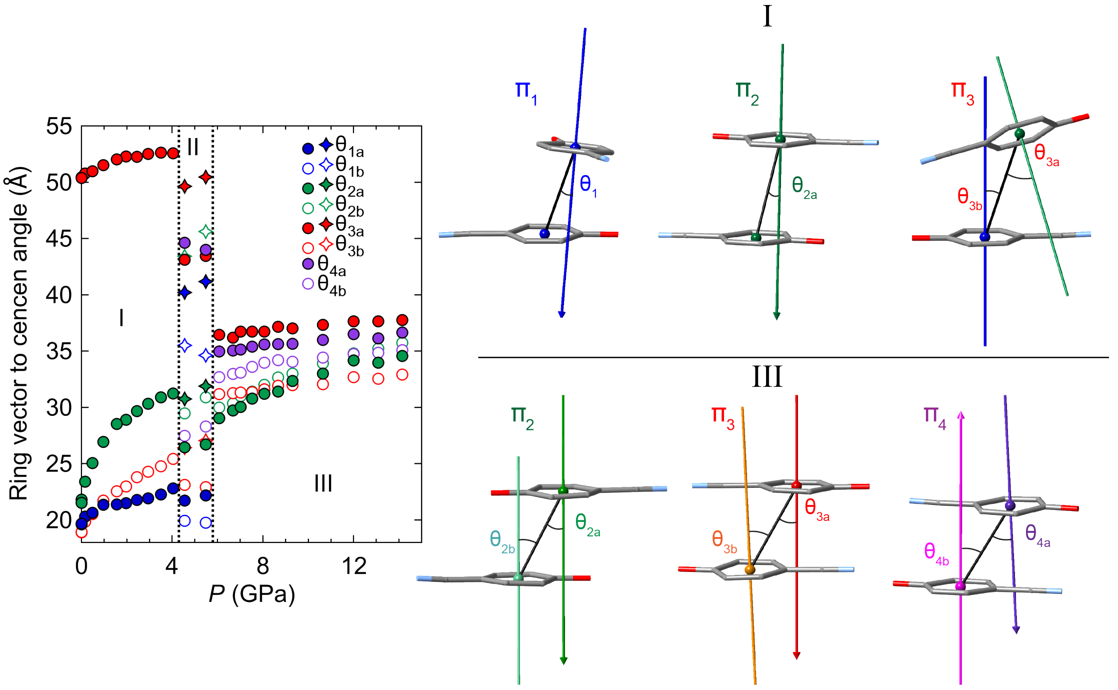

Figure S4. Angles between the benzenic ring normal and the centroid−centroid vector in 4HCB compressed in Ne as a function of pressure. The angles are illustrated for the three different combinations of interactions: centroid1−centroid1 (π_1), centroid2−centroid2 (π_2), and centroid2−centroid1 (π_3), shown at 0.15 GPa (phase I) and 6.1 GPa (phase III). The π_3 interaction from phase I becomes the π_3 and π_4 interactions in phase III. The abrupt changes in 4HCB orientations are observed upon the phase transition from phase I to III, in particular for the angle θ_{3a}. The orientations of 4HCB in phase II show intermediate values to phases I and III. The benzenic ring vectors are shown by the coloured arrows.

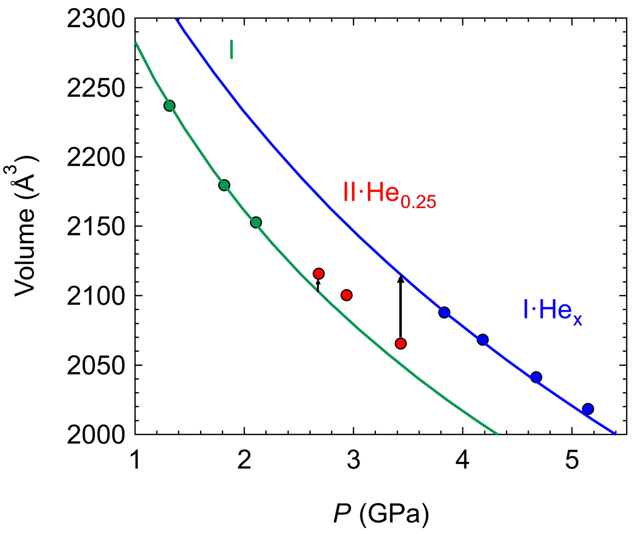

Figure S5. Zoom region of the pressure dependence of the volume of 4HCB compressed in helium with the two Vinet EoS fits shown extended beyond the phase boundaries to highlight the volume discontinuities upon the transitions from phases I to II·$He_{0.25}$ and from II·$He_{0.25}$ to I·He_x.

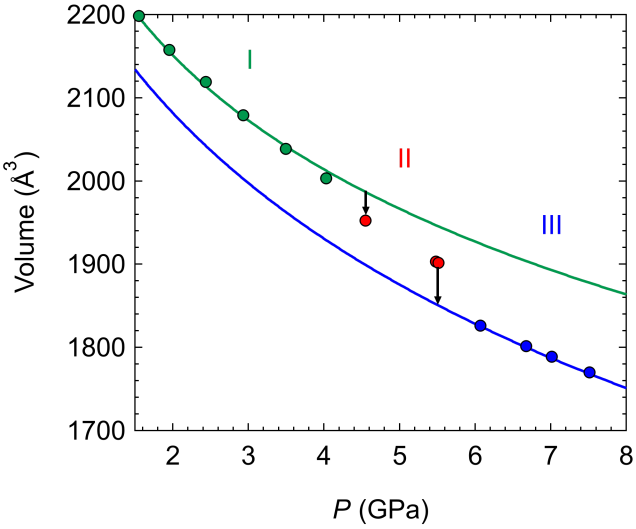

Figure S6. Zoom region of the pressure dependence of the volume of 4HCB compressed in neon with the two Vinet EoS fits shown extended beyond the phase boundaries to highlight the volume discontinuities upon the transitions from phases I to II and from II to III.

Table S1. Variable-pressure lattice parameters for 4HCB compressed in He. Decompression points are indicated by the asterisk.

s.g.	*P* (GPa)	*a* (Å)	*b* (Å)	*c* (Å)	*V* (Å^3)
Pbcn	0	9.1950(2)	10.785(10)	25.422(5)	2521(3)
Pbcn	0.38(9)	9.0335(11)	10.508(6)	25.355(3)	2406.7(13)
Pbcn	0.39(5)	9.0343(8)	10.506(5)	25.3489(19)	2406.0(11)
Pbcn	0.59(6)	8.9790(15)	10.385(10)	25.313(4)	2360(2)
Pbcn	0.89(6)	8.8912(5)	10.302(3)	25.1675(11)	2305.3(7)
Pbcn	1.32(6)	8.7981(7)	10.170(5)	24.9994(19)	2236.8(12)
Pbcn	1.82(6)	8.7104(5)	10.065(3)	24.8625(12)	2179.7(6)
Pbcn	2.11(7)	8.6612(8)	10.020(5)	24.805(2)	2152.7(12)
$Pbc2_1$	2.68(7)	8.5041(7)	10.013(5)	24.859(2)	2116.8(10)
$Pbc2_1$	2.94(5)	8.4470(6)	10.008(4)	24.8452(15)	2100.4(8)
$Pbc2_1$	3.43(5)	8.3793(7)	9.962(4)	24.7469(15)	2065.7(9)
Pbcn	3.83(7)	8.0103(6)	10.138(4)	25.7124(16)	2088.1(8)
Pbcn	4.18(7)	7.9689(5)	10.101(3)	25.6968(12)	2068.4(6)
Pbcn	4.67(8)	7.9340(5)	10.037(3)	25.6360(13)	2041.4(7)
Pbcn	5.15(8)	7.9066(3)	9.9794(17)	25.5818(7)	2018.5(3)
Pbcn	5.73(8)	7.8413(5)	9.918(3)	25.6095(11)	1991.6(6)
Pbcn	6.01(5)	7.8042(3)	9.8891(19)	25.6413(7)	1978.9(4)
Pbcn	6.68(9)	7.7102(3)	9.8329(17)	25.6882(6)	1947.5(3)
Pbcn	7.35(9)	7.6406(4)	9.773(2)	25.7208(10)	1920.6(5)
Pbcn	8.14(9)	7.5596(5)	9.724(3)	25.7447(10)	1892.5(6)
Pbcn	8.82(7)	7.5006(3)	9.6684(18)	25.7636(7)	1868.4(4)
Pbcn	9.51(10)	7.4407(2)	9.6127(15)	25.7669(6)	1843.0(3)
Pbcn	10.39(7)	7.3788(2)	9.5531(14)	25.7759(6)	1817.0(3)
Pbcn	11.59(7)	7.3101(3)	9.472(2)	25.7662(10)	1784.1(4)
Pbcn	13.16(9)	7.2279(2)	9.3720(16)	25.7111(7)	1741.7(3)
Pbcn	14.37(7)	7.1814(3)	9.302(2)	25.6646(9)	1714.4(4)
Pbcn	15.60(14)	7.14092(17)	9.2305(14)	25.6254(5)	1689.1(3)
Pbcn	17.06(8)	7.09190(17)	9.1593(13)	25.5615(5)	1660.4(2)
Pbcn	18.15(7)	7.06105(17)	9.1113(14)	25.5280(6)	1642.3(3)
Pbcn	19.73(8)	7.01610(15)	9.0454(12)	25.4792(5)	1617.0(2)
Pbcn	20.97(10)	6.9836(3)	8.989(2)	25.4326(9)	1596.6(4)
Pbcn	22.43(16)	6.9508(2)	8.9387(18)	25.3832(7)	1577.1(3)
Pbcn	24.01(9)	6.9149(3)	8.881(2)	25.3308(9)	1555.5(4)
Pbcn	25.05(8)	6.8912(2)	8.8456(18)	25.2918(7)	1541.7(3)
Pbcn	26.19(7)	6.8549(2)	8.8685(19)	25.2033(8)	1532.2(3)
Pbcn	24.93(8)*	6.8552(2)	8.9383(17)	25.2183(7)	1545.2(3)
Pbcn	20.34(9)*	6.9602(3)	9.089(3)	25.3824(12	1605.7(5)
Pbcn	15.98(16)*	7.0983(2)	9.241(17)	25.574(7)	1677.5(3)
Pbcn	15.29(12)*	7.1383(5)	9.251(5)	25.6569(9)	1694.3(2)
Pbcn	11.32(9)*	7.3154(7)	9.4877(4)	25.7595(9)	1787.9(2)
Pbcn	8.24(11)*	7.5379(5)	9.7108(3)	25.764(6)	1885.9(2)
Pbcn	6.30(6)*	7.7637(9)	9.8822(6)	25.6901(11)	1971.0(3)
Pbcn	4.94(9)*	7.9093(11)	10.004(7)	25.6199(14)	2027.2(3)
Pbcn	4.19(5)*	7.9494(16)	10.0965(11)	25.701(2)	2062.8(5)
Pbcn	3.57(9)*	8.0518(12)	10.1576(7)	25.6921(14)	2101.3(4)
Pbcn	2.46(12)*	8.5983(11)	10.0047(7)	24.8527(16)	2137.9(3)
Pbcn	1.50(11)*	8.7659(15)	10.1312(10)	24.999(2)	2220.1(5)
Pbcn	0.51(9)*	8.990(2)	10.4601(16)	25.347(3)	2383.6(7)

Table S2. Variable-pressure lattice parameters for 4HCB compressed in Ne. Decompression points are indicated by the asterisk.

s.g.	P (GPa)	a (Å)	b (Å)	c (Å)	V (Å^3)
Pbcn	0	9.236(9)	10.723(3)	25.435(6)	2519(3)
Pbcn	0.15(6)	9.103(3)	10.5907(8)	25.3998(19)	2448.9(7)
Pbcn	0.48(6)	8.983(7)	10.3997(16)	25.268(5)	2360.5(18)
Pbcn	0.96(5)	8.853(3)	10.2312(7)	25.0800(18)	2271.7(7)
Pbcn	1.55(6)	8.763(2)	10.0835(10)	24.8806(18)	2198.5(6)
Pbcn	1.96(6)	8.694(4)	10.0156(9)	24.780(2)	2157.6(11)
Pbcn	2.44(5)	8.652(4)	9.9368(10)	24.651(2)	2119.2(10)
Pbcn	2.93(6)	8.586(3)	9.8703(8)	24.535(2)	2079.2(9)
Pbcn	3.50(7)	8.521(3)	9.8026(8)	24.4071(19)	2038.7(8)
Pbcn	4.03(7)	8.464(4)	9.7418(8)	24.297(2)	2003.5(9)
$Pbn2_1$	4.55(8)	16.239(13)	9.7081(15)	24.772(4)	3905(3)
$Pbn2_1$	5.48(6)	16.094(8)	9.5959(10)	24.649(2)	3806.8(19)
$Pbn2_1$	5.52(7)	16.087(4)	9.6050(16)	24.614(2)	3803.2(11)
$Pbc2_1$	6.07(9)	7.254(3)	10.1150(11)	24.889(3)	1826.3(9)
$Pbc2_1$	6.68(9)	7.206(2)	10.0711(7)	24.828(2)	1801.7(6)
$Pbc2_1$	7.02(6)	7.173(4)	10.0553(11)	24.801(3)	1788.8(10)
$Pbc2_1$	7.52(7)	7.145(5)	10.0132(14)	24.742(4)	1770.1(12)
$Pbc2_1$	8.06(9)	7.098(4)	9.9748(10)	24.685(3)	1747.8(9)
$Pbc2_1$	8.67(10)	7.061(2)	9.9371(7)	24.6307(17)	1728.2(5)
$Pbc2_1$	9.31(9)	7.025(3)	9.8926(10)	24.555(3)	1706.4(9)
$Pbc2_1$	10.64(9)	6.943(2)	9.8333(7)	24.4533(16)	1669.4(6)
$Pbc2_1$	12.00(8)	6.885(3)	9.7749(8)	24.3468(18)	1638.4(7)
$Pbc2_1$	13.08(8)	6.8377(17)	9.7300(5)	24.2607(11)	1614.1(4)
$Pbc2_1$	14.14(7)	6.791(2)	9.6906(5)	24.1729(12)	1590.9(5)
$Pbc2_1$	13.55(5)*	6.807(3)	9.7046(10)	24.249(2)	1601.9(8)
$Pbc2_1$	9.97(9)*	6.971(3)	9.8546(10)	24.5417(18)	1685.9(8)
$Pbc2_1$	6.76(11)*	7.202(5)	10.0454(10)	24.781(3)	1793(1)
$Pbc2_1$	5.20(25)*	7.390(9)	10.190(2)	24.901(5)	1875(2)
$Pbn2_1$	4.45(10)*	16.282(2)	9.7189(6)	24.7682(10)	3919.5(5)
Pbcn	3.83(10)*	8.4787(14)	9.7720(7)	24.3455(15)	2017.1(4)

References

(S1) Cliffe, M.J.; Goodwin, A.L. PASCal: a principal-axis strain calculator for thermal expansion and compressibility determination. *J. Appl. Cryst.* **2012**, *45*, 1321–1329.

(S2) Badenhoop, J.K.; Weinhold, F. Natural steric analysis: Ab initio van der Waals radii of atoms and ions. *J. Chem. Phys.* **1997**, *107*, 5422–5432.
